# THE MEANINGS OF TRUST IN AFRICAN AMERICAN COMMUNITIES AND THEIR ASSOCIATION WITH PARTICIPATION IN DEMENTIA RESEARCH

**DOI:** 10.1093/geroni/igz038.1572

**Published:** 2019-11-08

**Authors:** Elena Portacolone, Peter Lichtenberg, Sahru Keiser, Leah Vest, Marsha Maloof, Julene Johnson

**Affiliations:** 1 University of California San Francisco, San Francisco, California, United States; 2 Wayne State University, Detroit, Michigan, United States; 3 UCSF, San Francisco, California, United States; 4 WSU, Detroit, Michigan, United States; 5 Pendergrass Smith, San Francisco, California, United States

## Abstract

African American /Black American older adults’ low participation in research reduces the generalizability of research findings and hinders understanding of dementia mechanisms, further widening health disparities. Both the Alzheimer’s Association and the National Institutes of Health have identified recruitment of African Americans with cognitive impairment into dementia research as an area of high priority. Distrust of research and medical institutions is often cited as a barrier to participation of African Americans in dementia research. Therefore, the goal of this study is to better understand African American community members’ expectations associated with trust. We used focus groups and semi-structured interviews to examine the expectations associated with overall trust. We conducted 6 focus groups: 4 with African American older adults and 2 with caregivers of African American older adults with cognitive impairment. We also interviewed 5 African American older adults with cognitive impairment (total n=59). Data were analyzed with content analysis. Five themes emerged: 1) Importance of providing truthful help/information leading to trust; 2) Long relationships leading to trust; 3) Acting efficiently and consistently (e.g., “not fooling around”) leading to trust; 4) Transference of trust (e.g., I can likely trust someone trusted by a trusted person); 5) Difficult to trust because of a harsh social environment. To conclude, trust is a complex belief associated with multiple expectations and relationships. It is critical that researchers understand these expectations related to trust in order to increase recruitment of African American older adults into dementia research.

